# Human liver microsomes study on the inhibitory effect of plantainoside D on the activity of cytochrome P450 activity

**DOI:** 10.1186/s12906-022-03671-5

**Published:** 2022-07-23

**Authors:** Jin Zhou, Xian Qian, Yanqing Zhou, Shili Xiong, Shuxia Ji, Ying Wang, Ping Zhao

**Affiliations:** 1 Department of Pharmacy, Shanghai Baoshan Luodian Hospital, Shanghai, 201908 China; 2 Clinical Research Center, Shanghai Baoshan Luodian Hospital, No.121 Luoxi Road, Baoshan District, Shanghai, 201908 China; 3 School of Pharmacy, Shanghai University of Traditional Chinese Medicine, 1200 Cailun Road, Shanghai, 201203 China

**Keywords:** CYP450 activity, Microsomes, Cocktail method, IC_50_, Time-dependent inhibition

## Abstract

**Background:**

Plantainoside D is widely existed in the herbs and possesses various pharmacological activities, making it possible to co-administrate with other herbs. Its effect on cytochrome P450 enzymes (P450) is a risk factor for inducing adverse drug-drug interactions. To assess the effect of plantainoside D on the activity of major P450 isoenzymes in human liver microsomes.

**Methods:**

The Cocktail method was conducted in human liver microsomes in the presence of probe substrates. The activity of P450 isoenzymes was evaluated by the production of corresponding metabolites. The concentration-dependent and time-dependent inhibition assays were performed in the presence of 0, 2.5, 5, 10, 25, 50, and 100 μM plantainoside D to characterize the inhibitory effect of plantainoside D.

**Results:**

Significant inhibition was observed in the activity of CYP1A2, 2D6, and 3A, which was concentration-dependent with the IC_50_ values of 12.83, 8.39, and 14.66 μM, respectively. The non-competitive manner and competitive manner were observed in the CYP3A inhibition (*Ki* = 7.16 μM) and CYP1A2 (*Ki* = 6.26 μM) and 2D6 inhibition (*Ki* = 4.54 μM), respectively. Additionally, the inhibition of CYP3A was found to be time-dependent with the *KI* of 1.28 μM^−1^ and *K*_*inact*_ of 0.039 min^−1^.

**Conclusions:**

Weak inhibitory effects of plantainoside D on the activity of CYP1A2, 2D6, and 3A were revealed *in vitro*, implying its potential of inducing interactions with CYP1A2-, 2D6-, and 3A-metabolized drugs. Although further *in vivo* validations are needed, the feasibility of the Cocktail method in evaluating P450 activity has been verified.

**Supplementary Information:**

The online version contains supplementary material available at 10.1186/s12906-022-03671-5.

## Background

Due to the increasing incidences and mortality of cardiovascular disease, great efforts have been devoted to improving clinical therapeutic and precaution strategies, among which traditional Chinese medicine has gradually ranked a critical position [1–3]. *Plantago asiatica* L*.* is a commonly used herb in the treatment of cardiovascular disease, which possesses the activity of preventing hyperlipidemia and atherosclerosis, two major risk factors of cardiovascular diseases [4–6]. Plantainoside D widely exists in various herbs, including *P. asiatica* L., which is mainly responsible for its pharmacological activities. Previous studies have demonstrated the protective effect of plantainoside D on cardiac muscle cells from the damage of adriamycin by suppressing ROS generation and NF-kB activation [7]. Plantainoside D was also considered to be one of the most promising inhibitors of IkB kinase-β and therefore exerted cardioprotective effects [8].

The potential interaction between different active ingredients of co-administrated herbs would induce adverse therapeutic effects and even toxicity. The liver is the main site of xenogeneic metabolism, including drugs, herbs, and also some toxics. The biotransformation processes in livers are primarily mediated by various enzyme systems in the microsomes, where cytochrome P450 enzymes (P450s) play vital roles [9]. Additionally, the function of P450s during the interactions has been publicly known and they are sensitive to environmental factors, especially the co-existing compounds [10–12]. Several active ingredients of different herbs were reported to exert effects on P450s activity. For example, Weiss et al. identified several fractions of clementines juice and found that the flavonoids could induce CYP3A4 and 1A2, which were inhibited by nobiletin, sinensetin, and tangeretin to varying degrees [13]. Clinically, adverse reactions caused by drug-drug interaction occupied an important position, where the affected activity of P450 isoenzymes is the most common mechanism [14, 15]. During the prescription of *P. asiatica* L. or other source herbs of plantainoside D, there would be a certain co-administration of other herbs with similar or auxiliary indications, which improves the potential of herb-herb interactions.

This study focused on the effect of plantainoside D on the activity of several P450 isoenzymes, including CYP1A2, 2A6, 2B6, 2C8, 2C9, 2C19, 2D6, 2E1, and 3A in human liver microsomes. The kinetic models and inhibition characteristics of affected isoenzymes were also evaluated, aiming to provide theoretical reference for the clinical prescription of plantainoside D original herbs or other CYP-metabolized herbs applicable for cardiovascular diseases.

## Methods

### Chemicals and reagents

Plantainoside D (Figure S1) was obtained from the National Institute of the Control of Pharmaceutical and Biological Products with a purity ≥ 98%. The specific substrates of the studied P450s, including phenacetion, paclitaxel, S-mephenytoin, burpropion, testosterone, coumarin, diclofenac, dextromethorphan, and chlorzoxazone were purchased from ICN Biomedicals and Sigma Chemical Co. The positive inhibitors of P450s, including furafylline (for CYP1A2), quinidine (for CYP2D6), and ketoconazole (for CYP3A) were purchased from Sigma Chemical Co. and Beijing Aleznova Pharmaceutical. The purity of the above reagents was no less than 98% and was of analytical reagent grade. Pooled human liver microsomes were obtained from BD Biosciences Discovery Labware.

### P450s assay

The activity of P450s was evaluated by corresponding metabolites using HPLC with Agilent 1260 Series Rapid Resolution HPLC. The reaction system was prepared with the following compositions: HLMs with certain protein concentrations, specific substrates of different isoenzymes, PBS buffer solution, plantainoside D or specific inhibitors, and an NADPH generating system as previously reported [16, 17]. Besides dextromethorphan and quinidine were dissolved in water, other substrated, inhibitors, and plantainoside D were dissolved in methanol with a final concentration of 1% (v/v). The volume of the reaction system was 200 μL and the reaction conditions are summarized in Table 1. The HPLC conditions are summarized in Table 2. Before initiating the reaction, there was a preincubation of 3 min followed by the addition of the NADPH-generating system.

**Table 1:** Isoforms tested, marker reactions, incubation conditions, and K_m_ used in the inhibition study

CYPs	Marker reactions	Substrate concentration (μM)	Protein concentration (mg/mL)	Incubation time (min)	Estimated K_m_ (μM)	Inhibitor concentration (μM)	Inhibitors	Reference
1A2	phenacetin *O*-deethylation	40	0.2	30	48	10	furafylline	[16, 17]
2A6	coumarin 7-hydroxylation	1.0	0.1	10	1.5	10	tranylcypromine	[16, 17]
2B6	Bupropion to hydroxybuptopion	40	0.25	30	50	50	monoterpenoid	[18, 19]
2C8	paclitaxel 6α-hydroxylation	10	0.5	30	16	5	montelukast	[16, 17]
2C9	diclofenac 4’-hydroxylation	10	0.3	10	13	10	sulphaphenazole	[16, 17]
2C19	*S*-Mephenytoin 4-hydroxylation	100	0.2	40	105	50	tranylcypromine	[16, 17]
2D6	dextromethorphan *O*-demethylation	25	0.25	20	4.8	10	quinidine	[20]
2E1	chlorzoxazone 6-hydroxylation	120	0.4	30	126	50	clomethiazole	[16, 17]
3A4	testosterone 6β-hydroxylation	50	0.5	10	53	1	ketoconazole	[16, 17]

**Table 2:** The detection conditions of HPLC for each CYP450 isoenzymes

cytochromes	Internal reference	Mobile phase	wavelength
CYP1A2	7-Hydroxycoumarin	Methanol: phosphate buffer (pH = 3.0, 50 mM) = 32:68	UV 245 nm
CYP2A6	-	Acetonitrile: acetic acid (0.1%, v/v) = 35:65	Fluo Ex/EM 340/456 nm
CYP2B6	Hydroxybupropion	Acetonitrile: water (v/v) = 20:80 for 2.5 min, 90–10 for 4 min	UV 268 nm
CYP2C8	-	Methanol: Water = 65:35	UV 230 nm
CYP2C9	Coumarin	Acetonitrile (A): phosphate buffer (pH = 7.4, 100 mM, B) = 32:68, 0–9 min, 68% B-32% B	UV 280 nm
CYP2C19	Tolbutamide	Methanol: potassium phosphate (pH 7.0, 10 mM) = 30:70	UV 204 nm
CYP2D6	-	Acetonitrile: phosphate buffer (pH = 3.0, 50 mM) = 25:75	Fluo Ex/EM 235/310 nm
CYP2E1	Phenacetin	Acetonitrile: acetic acid (0.5%, v/v) = 22:78, 1–10 min, 78% B-40% B	UV 287 nm
CYP3A4	Corticosterone	Methanol: water = 50:40, 0–15 min, 48% B-30% B; 15–22 min, 30% B-20% B	UV 254 nm

### Concentration-dependent inhibition and inhibition model fitting analysis

The effect of plantainoside D was primarily evaluated with the concentration of 0, 2.5, 5, 10, 25, 50, and 100 μM and the values of IC_50_ was evaluated. Furthermore, the inhibition model of P450 isoenzymes was estimated in the presence of various concentrations of corresponding substrates and fitting with Lineweaver–Burk and Dixon plots, and the inhibition constant was calculated by the following equations:

$v=\left(V_{max}S\right)/\left[K_m\left(1+\mathrm I/K_{\mathit i}\right)+S\right]$ for competitive inhibition;

$v\:=\:(V_{max}S)/\lbrack K_m\:+\:S(1\:+\:\mathrm I/K_iK_I)\rbrack$ for non-competitive inhibition.

where *I* is the concentration of plantainoside D, *K*_*i*_ is the inhibition constant, *S* is the concentration of the substrate, and *K*_*m*_ is the substrate concentration at half the maximum velocity (*V*_*max*_) of the reaction.

### Time-dependent inhibition

A total of 1 mg/mL HLMs were firstly incubated with 20 μM plantainoside D and the NADPH-generating system for 30 min. Then, incubation with 20 μL aliquot was carried out for 0, 5, 10, 15, and 30 min in the presence of substrates concentration close to *K*_*m*_. Additionally, the values of *KI* and *K*_*inact*_ were evaluated in the presence of substrates approximate to fourfold *K*_*m*_ according to the equation: $1/\mathrm{Kobs}\:=\:KI/K_{inact}\:\times\:1/\lbrack\mathrm I\rbrack\:+\:1/K_{inact}$.

### Statistical analysis

The comparison was performed with the student’s t-test using SPSS 26.0 software. *P* < 0.05 indicates statistical significance. The correlated fitting analyses were performed with Graphpad Prism 7.0 software.

## Results

### Plantainoside D significantly inhibited P450 activity in a concentration-dependent manner

**Fig. 1:**
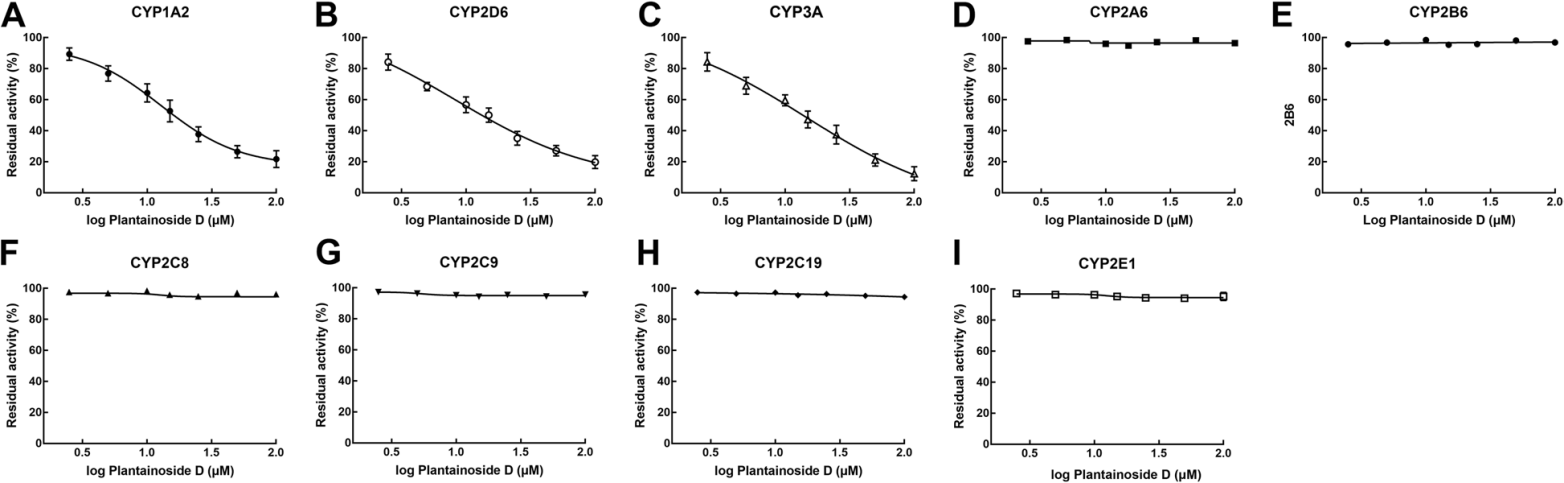
Concentration-dependent evaluation of CYP1A2 (**A**), 2D6 (**B**), 3A (**C**), 2A6(**D**), 2B6 (**E**) 2C8 (**F**), 2C9 (**G**), 2C19 (**H**), and 2E1 (**I**). The activity of CYP1A2, 2D6, and 3A was decreased with the increasing concentration of plantainoside D

**Fig. 2:**
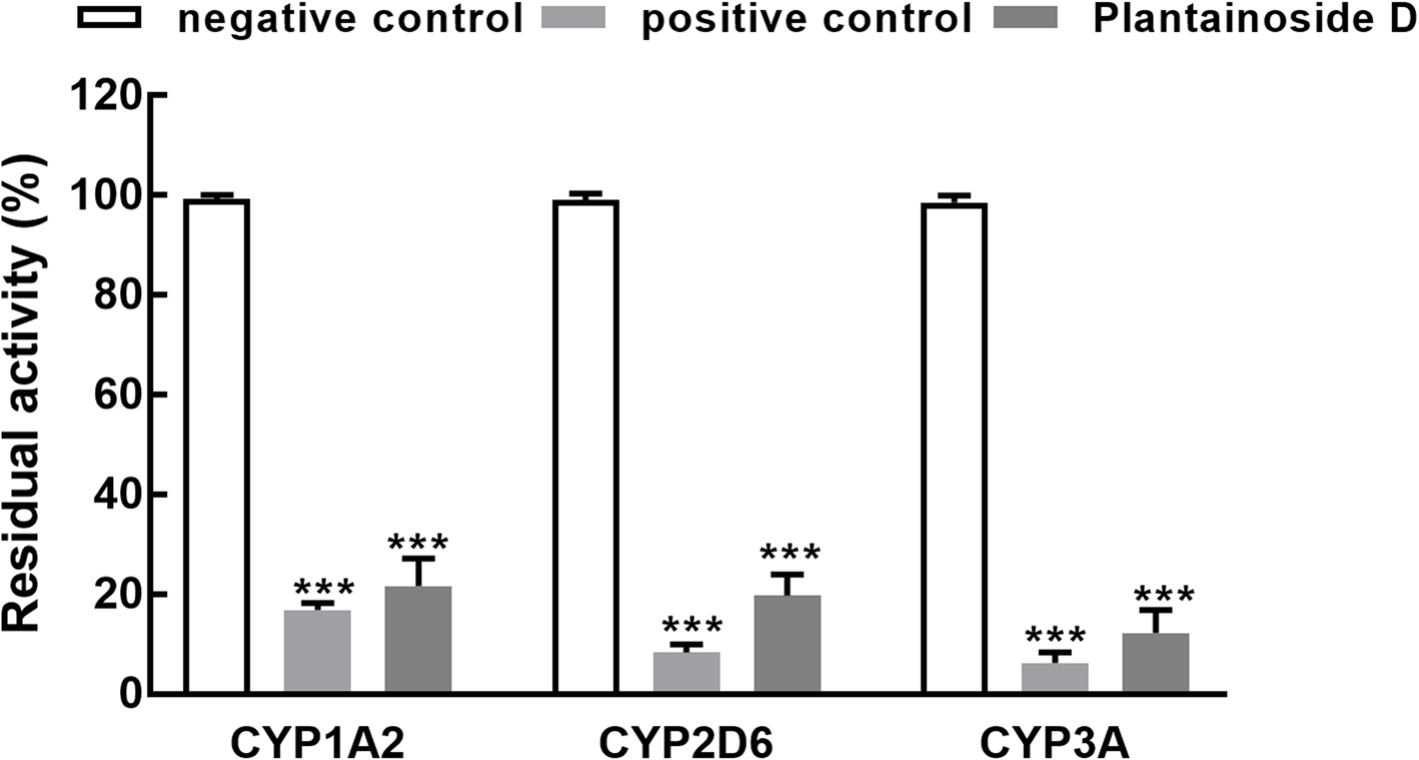
The residual activity of CYP1A2, 2D6, and 3A in the presence of specific inhibitors (positive control) or 100 μM plantainoside D with untreated HLMs as the negative control. The inhibitory effect of plantainoside D was relatively weaker than corresponding inhibitors. ^***^P < 0.001 compared with negative control

**Fig. 3:**
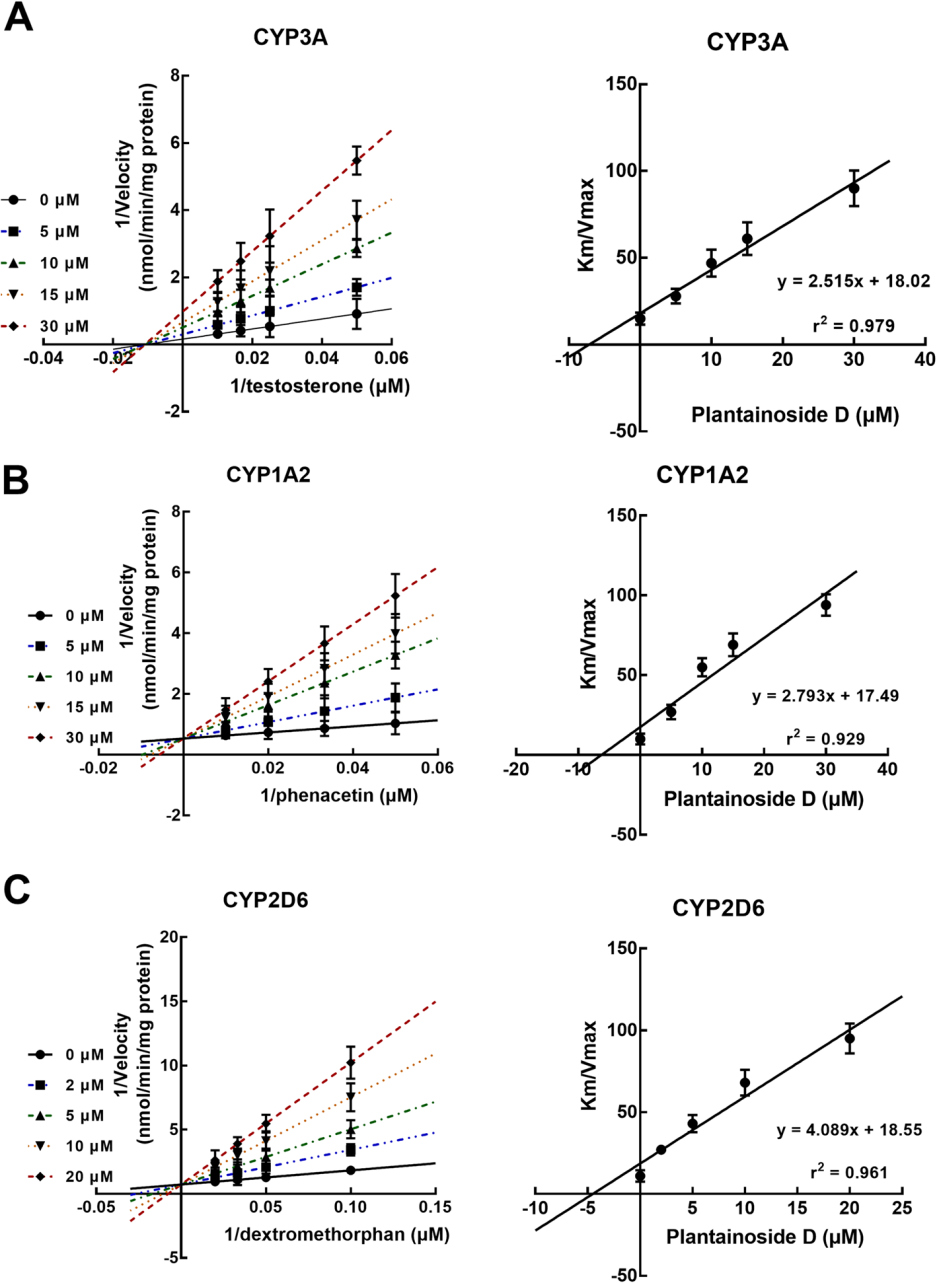
Inhibition model fitted with Lineweaver–Burk and Dixon plots. **A** The inhibition of CYP3A was best fitted with the non-competitive inhibition model and the Ki value was obtained as 7.16 μM according to the Dixon plot. **B**-**C** The inhibition of CYP1A2 (**B**) and CYP2D6 (**C**) was best fitted with the competitive inhibition model with the Ki values of 6.26 and 4.54 μM, respectively, according to the results of Dixon plots

In the presence of 0, 2.5, 5, 10, 25, 50, and 100 μM of plantainoside D, the activity CYP1A2 was suppressed, and the activity decreased with the concentration elevated with the IC_50_ value of 12.83 μM (Fig. 1A). Similar inhibitory effect and concentration-dependent manner was also observed in the activity of CYP2D6 (IC_50_ = 8.39 μM, Fig. 1B) and 3A (IC_50_ = 14.66 μM, Fig. 1C). While the activity of other isoenzymes, including CYP2A6 (Fig. 1D), 2B6 (Fig. 1E), 2C8 (Fig. 1F), 2C9 (Fig. 1G), 2C19 (Fig. 1H), and 2E1 (Fig. 1I), showed no changes in the presence of plantainoside D. Additionally, the inhibitory effect of plantainoside D was found to be relatively weaker than that of specific inhibitors on corresponding isoenzymes (Fig. 2).

### Inhibition model of CYP3A, 1A2, and 2D6

The result of the Lineweaver–Burk plot showed that the inhibition of CYP3A by plantainoside D was best fitted with the non-competitive inhibition model with the constant *K*_*m*_ (Fig. 3A). Furthermore, the Ki value of the non-competitive inhibition of CYP3A was obtained to be 7.16 μM according to the Dixon plot (Fig. 3A).

Both the inhibition of CYP1A2 and 2D6 were demonstrated to be best fitted with a competitive inhibition model with a constant *V*_*max*_ (Fig. 3B and C). The Ki values of CYP1A2 and 2D6 were 6.26 and 4.54 μM, respectively, according to the results of Dixon plots.

### Time-dependent manner evaluation

**Fig. 4:**
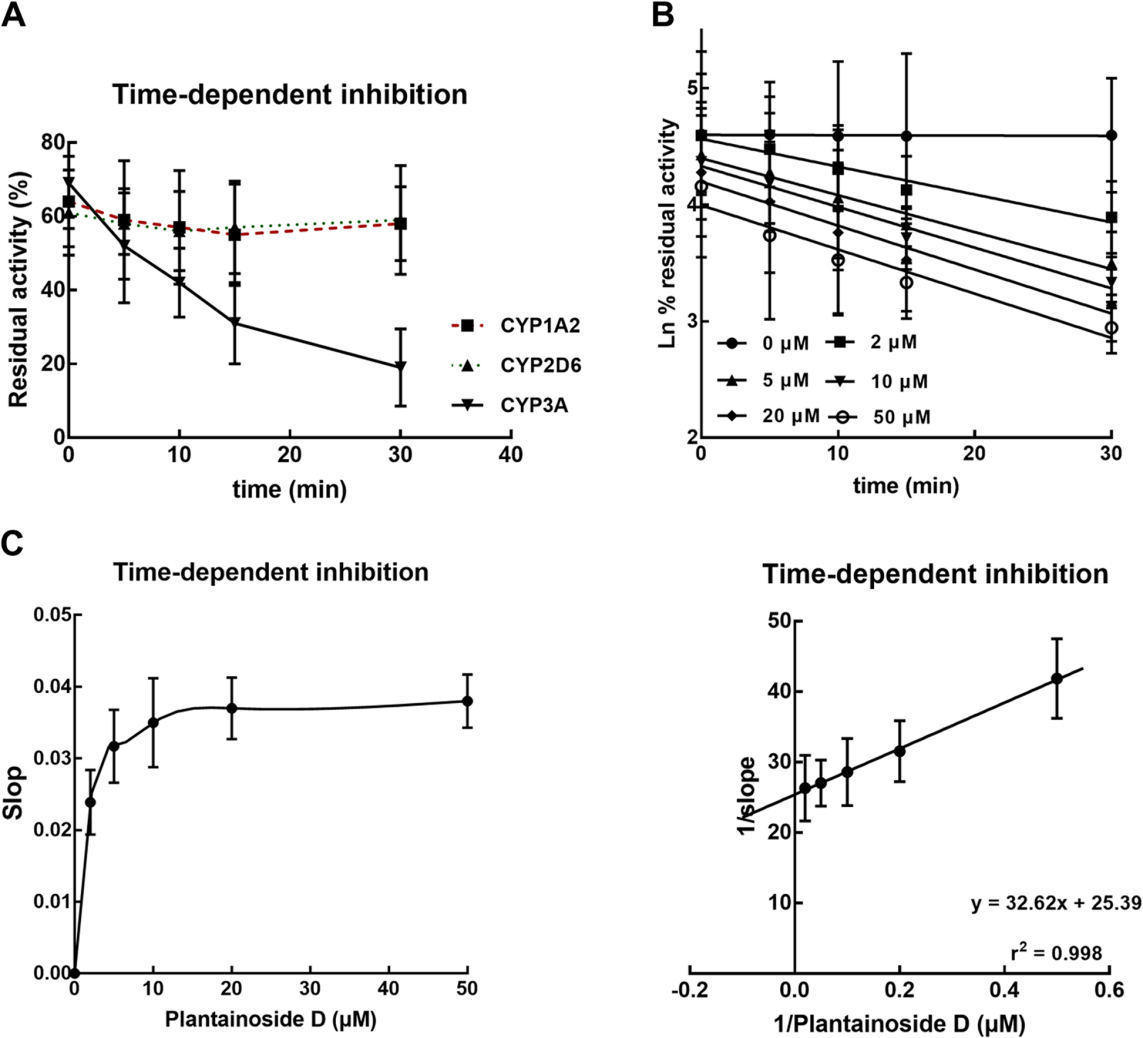
Time-dependent evaluation. **A** The inhibition of CYP3A was found to be time-dependent, whereas CYP1A2 and CYP2D6 was not affected by incubation time. **B**-**C** Time-dependet inhibition of CYP3A in the presence of 0, 2, 5, 10, 20, and 50 μM plantainoside D (**B**). The values of KI and Kinact were calculated as 1.28 and 0.039 (**C**)

The inhibitory effect of plantainoside D on CYP3A was enhanced with the increasing incubation time, while the inhibition of CYP1A2 and 2D6 showed no significant changes with time (Fig. 4A). The time-dependent manner of CYP3A inhibition was observed in the presence of various concentrations of plantainoside D (Fig. 4B). The values of *KI* and *K*_*inact*_ were calculated as 1.28 and 0.039 (Fig. 4C).

## Discussion

Due to the wide involvement of P450s in the metabolism processes of various drugs, herbs, and other kinds of xenobiotics, the changes in their activity would significantly affect the therapeutical efficiency or even induce toxicity. Because of the limited sources and the poor repeatability of liver cells and several factors that would complex the P450 enzyme kinetics, liver microsomes are usually used to assess the inhibition characteristics of P450 isoenzymes and drug metabolism [21]. The present study investigated the effect of plantainoside D on the activity of eight major P450 isoenzymes in human liver microsomes, which are responsible for the biotransformation of 80% of drugs [22, 23]. The Cocktail method was carried out in the presence of various probe substrates evaluating P450 activity based on the production of metabolites, which could avoid individual differences and improve analytical efficiency [24, 25]. A significant inhibitory effect of plantainoside D was observed on the activity of CYP1A2, 2D6, and 3A. Although the inhibitory effect of plantainoside D was weaker than corresponding specific inhibitors, the observed results still indicated its great potential for interacting with CYP1A2-, 2D6-, and 3A-metabolizing drugs.

The inhibition of CYP1A2, 2D6, and 3A were all found to be concentration-dependent, which was enhanced by the increasing plantainoside D concentrations, and corresponding IC_50_ values were obtained. IC_50_ is a critical reference that represents the inhibition degree and guides the prescription of plantainoside D. Previously, IC_50_ < 100 μg/mL was considered a strong inhibition, while IC_50_ > 100 μg/mL was a weak inhibitory effect [26]. The inhibitory effects of plantainoside D on CYP1A2, 2D6, and 3A might induce herb-herb interactions. However, liver microsomes cannot simulate the *in vivo* physiological situation. The hepatic concentration of plantainoside D is a critical factor determining whether the inhibition of P450s occurs and how the degree is. Therefore, the specific interaction needs further clinical validation.

The non-competitive model was best fitted with the inhibition of CYP3A, while the competitive model was best fitted with CYP1A2 and 2D6 inhibition. Both non-competitive inhibition and competitive inhibition are reversible, which can be reversed or weakened by increasing the concentration of substrates [27]. The inhibition model could help the clinical dose of plantainoside D or plantainoside D-containing herbs and improve the safety and science of medication. CYP3A accounts for a huge proportion of the P450 family and has been illustrated to mediate drug-drug interactions. Among the herbs with similar indications of plantainoside D, several herbs or compounds were reported to be metabolized by CYP3A, which are of great potential to lead to adverse interactions [28–30]. The inhibition of CYP3A by plantainoside D was found to be time-dependent, and behaved as the reducing activity of CYP3A with prolonged incubation time. The competitive manner or the time-dependent manner was previously reported to relate to functional groups, such as aromatic and ethynyl groups, which can be found in plantainoside D [31, 32].

## Conclusions

Taken together, plantainoside D served as a non-competitive inhibitor of CYP3A and competitive inhibitor of CYP1A2 and 2D6. The inhibitory effect of plantainoside D was concentration-dependent and time-dependent. The potential of plantainoside D or its source herbs interacting with co-administrated herbs or drugs needs further *in vivo* investigations.

## Supplementary Information

**Additional file 1:** **Fig S1.** The chemical structure of plantainoside D.

## Data Availability

The datasets used and/or analysed during the current study are available from the corresponding author on reasonable request.
