# Supplementary figures and images for: Human liver microsomes study on the inhibitory effect of plantainoside D on the activity of cytochrome P450 activity
Source: BMC Complement Med Ther. 2022 Jul 23;22:197. doi: 10.1186/s12906-022-03671-5 (PMC9308932; doi:10.1186/s12906-022-03671-5)

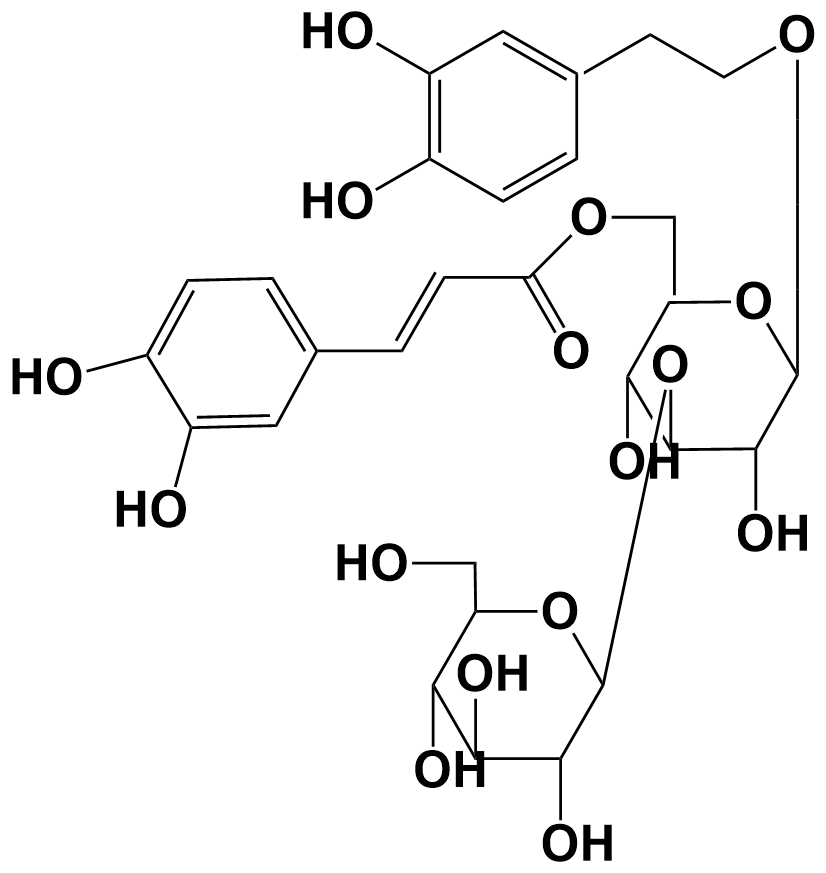

Supplement: Supplementary file 1 — Additional file 1: Fig S1. The chemical structure of plantainoside D. [file 12906_2022_3671_MOESM1_ESM.tif]
